# Oceanic Breakthroughs: Marine-Derived Innovations in Vaccination, Therapy, and Immune Health

**DOI:** 10.3390/vaccines12111263

**Published:** 2024-11-08

**Authors:** Chiara Gamberi, Chad L. Leverette, Alexis C. Davis, Moayad Ismail, Ilaria Piccialli, Nicola Borbone, Giorgia Oliviero, Caterina Vicidomini, Rosanna Palumbo, Giovanni N. Roviello

**Affiliations:** 1 Department of Biology, Coastal Carolina University, Conway, SC 29526, USA; cgamberi@coastal.edu (C.G.); cleverett@coastal.edu (C.L.L.); acdavis8@coastal.edu (A.C.D.); 2 Faculty of Medicine, European University, 76 Guramishvili Ave., 0141 Tbilisi, Georgia; mouaiadismail123@gmail.com; 3 Division of Pharmacology, Department of Neuroscience, Reproductive and Dentistry Sciences, School of Medicine, University of Naples Federico II, Via Pansini 5, 80131 Naples, Italy; 4 Department of Pharmacy, University of Naples Federico II, Via Domenico Montesano 49, 80131 Naples, Italy; borbone@unina.it; 5 Department of Molecular Medicine and Medical Biotechnologies, University of Naples Federico II, Via Sergio Pansini 5, 80131 Naples, Italy; 6 Institute of Biostructures and Bioimaging, Italian National Research Council (IBB-CNR), Via P. Castellino 111, 80131 Naples, Italy; caterina.vicidomini@ibb.cnr.it (C.V.); rosanna.palumbo@cnr.it (R.P.)

**Keywords:** marine microalgae, recombinant vaccines, *Nannochloropsis* spp., *Dunaliella salina*, immunomodulatory agents, vaccine adjuvants

## Abstract

The vast, untapped potential of the world’s oceans is revealing groundbreaking advancements in human health and vaccination. Microalgae such as *Nannochloropsis* spp. and *Dunaliella salina* are emerging as resources for recombinant vaccine development with specific and heterologous genetic tools used to boost production of functional recombinant antigens in *Dunaliella salina* and *Nannochloropsis* spp. to induce immunoprotection. In humans, several antigens produced in microalgae have shown potential in combating diseases caused by the human papillomavirus, human immunodeficiency virus, hepatitis B virus, influenza virus, Zika virus, Zaire Ebola virus, *Plasmodium falciparum*, and *Staphylococcus aureus*. For animals, microalgae-derived vaccine prototypes have been developed to fight against the foot-and-mouth disease virus, classical swine fever virus, vibriosis, white spot syndrome virus, and *Histophilus somni*. Marine organisms offer unique advantages, including the ability to express complex antigens and sustainable production. Additionally, the oceans provide an array of bioactive compounds that serve as therapeutics, potent adjuvants, delivery systems, and immunomodulatory agents. These innovations from the sea not only enhance vaccine efficacy but also contribute to broader immunological and general health. This review explores the transformative role of marine-derived substances in modern medicine, emphasizing their importance in the ongoing battle against infectious diseases.

## 1. Introduction

The world’s oceans are being increasingly regarded as an untapped treasure trove of bioactive compounds and organisms with remarkable therapeutic potential. Marine microalgae are common microscopic organisms growing as single cells, colonies, or extended filaments. Microalgae represent the largest primary biomass in the marine ecosystem and are emerging as key players in addressing global challenges such as climate change, energy shortages, and food insecurity. They also serve as a potent source of pharmaceuticals and bioactive compounds [1]. Microalgae [2,3] *Nannochloropsis* spp. and *Dunaliella salina* [4] are used for recombinant vaccine development. *Arthrospira platensis* is edible and a rich source of valuable secondary metabolites with medicinal properties. Microalgae are recognized as a significant source of nutritional supplements and metabolites for use in the pharmaceutical and nutraceutical industries [1]. These metabolites display various bioactivities, including anti-proliferative, anti-oxidative [5], pro-apoptotic, and anti-aging. Microalgal cell-free extracts have been explored as antimicrobial additives in food and feed formulations to reduce reliance on synthetic preservatives and combat antimicrobial resistance.

### Microalgae and Vaccines

*D. salina* is a green marine microalga belonging to the *Chlorophyceae* family, known for its extreme salt tolerance and ability to thrive in salinities ranging from 0.05 to 5.0 M NaCl [6]. It can maintain low intracellular sodium concentrations, making it ideal for cultivation in high-salinity environments that reduce contamination risks. *D. salina* lacks rigid cell walls, instead possessing a thin elastic membrane that allows rapid growth and high biomass productivity combined with the accumulation of valuable carotenoids and proteins under stress conditions. It was the first microalgal species commercially used for β-carotene production and has additional applications in bioethanol fermentation and biogas production [7]. *Nannochloropsis salina* is renowned for its high lipid content, particularly its ability to stimulate lipid accumulation upon nitrogen and nutrient starvation stress [6]. It plays a significant role in global carbon and mineral cycles in oligotrophic seawater. *N. salina* is used as a feed source in aquaculture and is considered a potential alternative to fish oil because of its high content of polyunsaturated fatty acids, particularly eicosapentaenoic acid. Its lipid production potential also makes it a promising candidate for biofuel development. Both *D. salina* and *N. salina* are used for various biotechnological applications, including vaccine production, due to their adaptability, sustainable production, and ability to produce bioactive compounds and complex antigens under controlled culture conditions [6]. Apart from microalgae, the ocean provides an array of innovative bioactive compounds that serve as potent adjuvants for vaccines, delivery systems, and immunomodulatory agents that contribute to broader immunological health [8,9]. Live recombinant vaccines express antigens in target cells and use genetically engineered live bacteria or viruses to elicit immune responses against a pathogen. They offer both safety and long-lasting protection by combining the best features of recombinant and attenuated vaccines. Interestingly, some of these vaccines are administered orally or nasally, such as the nasal spray influenza vaccine and the oral polio vaccine [10,11]. Another important advancement is the realization that so-called aquafoods, such as fish, mollusks, crustaceans, and seaweeds, effectively enhance natural immunity due to their diverse and rich content of biofunctional compounds and thereby complement the efficacy of live recombinant vaccines [11]. In fact, aquafoods are abundant in essential nutrients such as proteins, amino acids, omega-3 polyunsaturated fatty acids, vitamins, minerals, and pigments that promote immune competence [12] and help protect against infections [11]. Research into these immune-boosting properties highlights their potential for disease prevention and improved public health. Understanding the optimal daily intake of these compounds could further improve dietary recommendations for enhanced immunity and reduced risk of infectious diseases [12]. Sepsis, a life-threatening condition resulting from a dysregulated immune response to infection, often stems from exposure to bacterial components, particularly lipopolysaccharides (LPS) found in the outer membranes of Gram-negative bacteria [13]. The lipid A portion of LPS is vital for this immune activation, and alterations to its structure can dramatically affect immune responses. Mass spectrometry-based structural analysis has revealed that bacteria often modify their LPS structures in response to environmental changes or during host infection to escape immune detection and response [14]. Interestingly, certain marine bacteria, particularly those isolated from deep-sea environments (e.g., *Moritella* spp.) exhibit immunosilence, possibly because of the length of the acyl chain in their LPS. Therefore, uncovering novel microbial interactions may inform both immunology and therapeutic development [15]. In summary, the vast untapped potential of marine ecosystems is yielding transformative advancements in the field of vaccination and immune health. Marine-derived innovations, particularly from microalgae, offer significant promise in developing sustainable, effective vaccines. These organisms’ ability to produce complex antigens and bioactive compounds under controlled conditions highlights their role in advancing modern vaccine technologies. Including biofunctional compounds from aquatic species enhances vaccine efficacy and contributes to broader immune support, demonstrating the critical role of marine environments in human health. This review focuses on the specific contributions of marine bioresources to vaccine strategies and immune system modulation, discussing their immediate relevance to both human and animal health applications.

## 2. Microalgae-Based Recombinant Vaccines

**Scheme 1:**
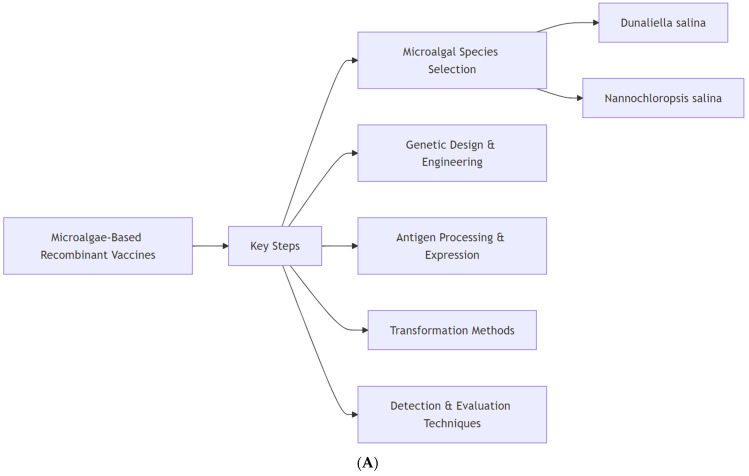

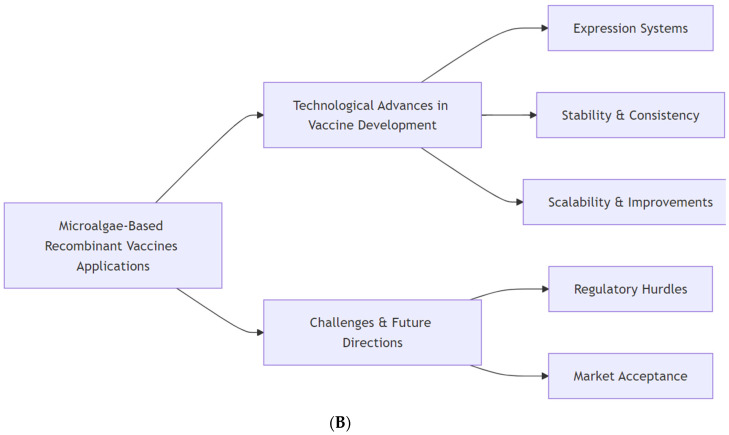
Overview of microalgae-based recombinant vaccines. (**A**): Fundamental steps in the development of microalgae-based recombinant vaccines begin with the selection of an appropriate microalgal species, often *Dunaliella salina* and *Nannochloropsis salina*, which are recognized for their biotechnological potential. Genetic design and engineering define the features of antigen expression and processing and the transformation method. Finally, the expressed antigen is detected, and its vaccine efficacy is detected. (**B**): Microalgae-based recombinant vaccines have spurred technological innovations in expression systems, improved stability and consistency, and robust scalability and improvements that enhance commercial viability. Microalgae-based technology in vaccine production could provide effective and sustainable solutions for both human and animal health. Regulatory hurdles and possibly low market acceptance represent challenges to overcome that currently limit the widespread adoption of these novel vaccines.

Vaccination is a key method for controlling and preventing many infectious diseases and toxic reactions prior to encountering the responsible pathogen or toxin [16,17,18,19,20,21]. Vaccination relies on the active presentation of antigens to stimulate an immune response. Vaccines can be categorized into live attenuated vaccines, such as those for smallpox and measles, recombinant subunit vaccines, including those for hepatitis B, among others, and RNA vaccines, such as the one for COVID [22,23,24,25,26]. The first proof-of-concept study for microalgae-based vaccines was published nearly two decades ago, and various biopharmaceuticals have been produced since then [9]. Scheme 1 illustrates the primary elements involved in the development and evaluation of vaccines using genetically engineered microalgae, including species selection, antigen processing, transformation methods, and detection techniques. It also outlines the potential applications, technological advances, and challenges associated with this approach.

### 2.1. Development of Microalgae-Based Recombinant Vaccines

Researchers developing microalgae-based vaccines for infectious diseases carefully consider numerous factors, including selecting the appropriate microalgal species based on their biological traits, designing the genetic construct with attention to antigen choice, expression vector, codon optimization, and antigen glycosylation, as well as determining whether the antigen should be secreted, encapsulated, or retained within subcellular compartments. Other critical considerations include the method of genome transformation (whether in the nucleus or plastids and whether it should be stable or transient), and transformation protocols. Furthermore, transgene and antigen detection need to be optimized, and the immunization strategy and administration route planned—injection of purified recombinant antigens, oral delivery using freeze-dried microalgae, or a combination of both—along with evaluating protective efficacy through experimental challenges with the target pathogen to ensure vaccine effectiveness. Although many algal species have been identified, only a few have been engineered for recombinant vaccine production. *Chlamydomonas reinhardtii* has been frequently used, largely due to the availability of genetic engineering tools. Recently, other species such as *D. salina* and *Nannochloropsis* sp., but also *Schizochytrium* sp., *Thalassiosira pseudonana*, and *Chlorella pyrenoidosa* have been utilized. These efforts have led to the production of functional recombinant antigens, and in some cases, the induction of immunoprotective responses in vaccinated individuals. Each microalgal species brings unique features that can be advantageous in producing recombinant vaccines [9]. The interest in using microalgae for this purpose resides in their ability to produce complex proteins and modify them post-translationally, and their sustainable large-scale culturing supporting low-cost production, which makes them an attractive alternative to traditional, expensive vaccine production methods [8]. Culturing in controlled environments ensures consistent quality and yields of recombinant proteins [27]. Chloramphenicol at a concentration of 60 μg mL^−1^ was found to stop algal growth, providing a basis for antibiotic resistance selection in genetic transformation. Moreover, the ubiquitin promoter showed the highest expression of a downstream gene. Using this system, the hepatitis B virus surface antigen (*HBsAg*) gene was successfully introduced into *D. salina* cells via electroporation and its genomic integration confirmed through PCR and Southern blot analyses, while the stable expression of the HBsAg protein was verified using enzyme-linked immunosorbent assay (ELISA) and Western blot analysis [27]. Remarkably, both the transgene and HBsAg expression remained stable for at least 60 generations in a chloramphenicol-free medium, underscoring the robustness of this system for long-term protein production [27]. Antigens produced in microalgae have elicited immune responses against several viruses, including HPV, Hepatitis B, and Zika in preclinical studies [28,29,30,31,32,33,34,35]. Microalgae also offer the potential for developing edible vaccines, where the antigen-expressing microalgae could be consumed to elicit the immunization response. Edible vaccines could simplify vaccination and increase accessibility, especially in resource-limited settings. They could elicit mucosal immunity, in which a “barrier immunity” is raised at the mucosal-associated lymphoid tissue (MALT) [36] to intercept the pathogen before it enters the body. However, the biggest challenge for edible vaccines remains to protect the antigen from degradation by the digestive enzymes. In this respect, the recombinant antigens produced in microalgae are protected by the algal cell wall and released at the MALT upon digestion. Although large amounts of recombinant cells are needed for oral vaccine administration, freeze-drying technology can enhance antigen concentration without losing its immunogenic properties. In addition to providing potential immunization, microalgae also produce “high-value” secondary metabolites with medicinal properties, such as antimicrobial and anti-cancer that may be beneficial as supplements or metabolic support [37,38]. Certain *Dunaliella* species contain immunomodulators that can work synergistically with the antigen. Additionally, microalgae have unique growth advantages, such as efficient photosynthesis and the ability to capture environmental CO_2_ [39,40], making them sustainable platforms for biotechnological applications. As previously mentioned, species like *D. salina* hold significant promise for recombinant protein production due to favorable traits such as ease of cultivation, natural encapsulation, and immunomodulatory compound production, with ongoing research into its genetic manipulation showing potential for developing scalable vaccine systems. Its ability to grow in high-salt media, combined with advances in genetic engineering techniques like codon optimization and vector design, positions *D. salina* as a cost-effective, industrial-scale platform for producing recombinant antigens, particularly for mucosal vaccine delivery, meeting the growing demand for low-cost, next-generation vaccines [41]. Mucosal immunity is particularly important for defense against pathogens as primary lymphoid organs and lymphocytic aggregates, such as the Harderian gland of the eye and the already cited MALT, contribute to local immune responses, especially through the production of secretory immunoglobulins (S-Igs) [42]. Apart from human vaccination, microalgae-based vaccines were developed to combat avian and animal diseases and prevent their spreading to humans, a phenomenon known as zoonosis [43]. In fact, by controlling animal diseases, vaccines can reduce the risk of some of these pathogens crossing over to humans [44,45].

### 2.2. Applications of Microalgae in Veterinary Vaccines and Aquaculture

In animals, microalgae-based vaccines have successfully targeted the foot-and-mouth disease virus, classical swine fever virus, white spot syndrome virus (WSSV), vibriosis, infectious bursal disease virus, and *Histophilus somni*, the pathogen causing cattle respiratory disease [9]. Microalgae-based vaccines could also be used to enhance immune responses in aquatic animals. *D. salina* was used to develop a subunit vaccine targeting WSSV, one of the most severe pathogens infecting crustacean species important for human consumption such as crayfish, shrimp, crabs, and lobsters. A transgenic *D. salina* expressing high levels of the bioactive WSSV VP28 protein was made and fed to crayfish, resulting in effective protection against WSSV. This breakthrough showcased the potential of *D. salina* as a vaccine production platform, offering a cost-effective and scalable solution for preventing viral diseases in aquaculture with potentially broad applications [46].

In the same context, avian influenza [47,48,49] remains a significant global threat to the poultry industry. Vaccination strategies focus on inactivated, live attenuated, virus and recombinant viral hemagglutinin (HA) protein as the primary component of these vaccine formulations. Subunit antigens have shown potential for inducing both local and systemic immune responses in poultry, particularly when administered via the ocular route involving the Harderian gland. Complex proteins, such as HA from the avian influenza virus H5N2, a relatively mild virus periodically spreading in commercial cultures and causing the culling of millions of infected birds, can effectively stimulate localized production of HA-specific S-Igs and generate a specific systemic immune response. Therefore, *D. salina*-produced HA-antigens may constitute a viable strategy for targeted vaccination in poultry [42], especially if administered through food. Similarly, the product of the *H5HA* gene from the H5N2 virus, a significant threat to both poultry and humans, was expressed in *D. salina* and found to be bioactive and immunogenic. These findings highlight the *D. salina* potential as an alternative biological platform for expressing complex viral antigens, such as HA5r from the H5N2 strain, offering a novel approach to avian influenza vaccine production through microalgal systems [50]. Apart from their use in antigen production for vaccination, microalgae have also demonstrated potential in enhancing innate immunity in aquaculture. Dietary supplementation with *Nannochloropsis oculata* over eight weeks has significantly improved growth, immune markers, and disease resistance in Nile tilapia (*Oreochromis niloticus*) juveniles against the bacterial pathogen *Aeromonas veronii*. Fish fed diets with 5% *N. oculata* showed significantly enhanced growth parameters, including final body weight, weight gain, specific growth rate, and total feed intake, while also reducing the feed conversion ratio compared to the control group. Fish supplemented with 15% *N. oculata* showed enhanced immune response reflected in a significant decrease in total protein, albumin, globulin, and the albumin/globulin ratio [51], while liver enzyme activities, including alanine transaminase and aspartate transaminase, remained unaffected. Immune markers such as serum lysozyme activity, nitric oxide, and nitroblue tetrazolium levels were significantly elevated in fish fed 5% *N. oculata* diets. Histomorphological analysis of hepatopancreatic and intestinal tissues revealed normal structures in fish fed diets supplemented with 5% *N. oculata*. Higher *N. oculata* levels (10% and 15%) led to significant upregulation of cytokines, including interleukin-1β, interleukin-8, interferon-γ, transforming growth factor-β, and tumor necrosis factor-α (TNF-α), along with downregulation of the antioxidant superoxide dismutase (SOD). Disease resistance to *A. veronii* was also improved upon 10% and 15% *N. oculata* supplementation, highlighting the beneficial role of *N. oculata* in promoting growth and enhancing immune responses in Nile tilapia, and its potential as a valuable dietary supplement in aquaculture [51]. The microalga *Nannochloropsis* sp. was used as a vaccine carrier to combat vibriosis in fish. Vibriosis is a common disease affecting aquatic organisms, particularly fish and shellfish, caused by bacteria from the *Vibrio* genus. Transgenic *Nannochloropsis* sp. was genetically modified to express a fragment of an outer membrane protein kinase (OmpK) gene from *Vibrio* species and was used as an oral vaccine for fish. The *OmpK* transgene remained present and active for several generations of transgenic *Nannochloropsis* sp., confirming its suitability as a vaccine carrier for aquaculture purposes [52]. While specific data on the immunomodulatory benefits of microalgae for human consumption are still emerging, some evidence suggests that consuming microalgae may also enhance innate immunity in humans [53,54,55]. In conclusion, vaccination is a vital tool for controlling infectious diseases by effectively presenting antigens to the immune system of several host species, including aquatic invertebrates and vertebrates of commercial importance and, potentially, also in humans. Microalgae-based vaccines have emerged as a promising sustainable, low-cost, and scalable platform in the vaccination field.

## 3. Marine Natural Products: Emerging Bioactive Compounds and Their Potential as Vaccine Adjuvants and Therapeutics

Adjuvants, substances that boost the immune response to an antigen, are critical in vaccine formulation. Different marine-derived products have been shown to enhance vaccine efficacy [56,57,58,59] and the exploration of marine-derived adjuvants represents a promising frontier in vaccine development. Marine-derived secondary metabolites, with unique chemical structures and diverse biological activities, may also improve vaccine efficacy through desirable anti-inflammatory and antioxidant properties. COVID-19, caused by the outbreak of the SARS-CoV-2 virus that resulted in a global health crisis, causes systemic infections featuring tissue-damaging cytokine storms. This prompted the search for the discovery and integration of various molecular and therapeutic approaches, including vaccines, antivirals of synthetic and herbal origin, and immune-modulating treatments. Additions such as forest bathing and other nature-connected practices have also been explored to enhance physical and mental well-being, reduce stress, and boost immune function during the pandemic [60,61,62,63,64,65,66,67,68,69,70,71]. By harnessing the unique properties of these marine compounds, it is possible to create vaccines that elicit stronger and possibly more durable immune responses while reducing tissue damage and protecting against oxidative stress, potentially leading to better protection against a wide range of diseases. Polysaccharides, proteins, and lipids extracted from marine algae, sponges, and mollusks have shown promise as potent adjuvants, with one key example being fucoidan [72], a sulfated polysaccharide from brown seaweed, with demonstrated immunostimulatory properties. Fucoidan can enhance both humoral and cellular immune responses, making it an ideal candidate for a vaccine adjuvant. Sulfated galactans found in red algae [73,74,75] are also emerging as potent adjuvants with potential to enhance immune responses and provide antiviral protection.

### 3.1. Marine-Derived Polysaccharides as Potent Vaccine Adjuvants and Antiviral Agents

**Figure 1:**
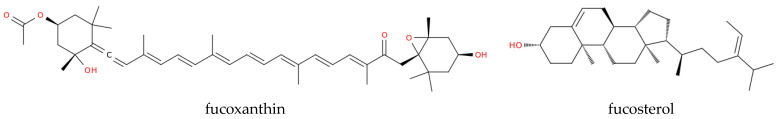
Chemical representation of fucoxanthin and fucosterol.

Sulfated galactans are linear polymers with alternating β-D-galactopyranose and α-D-galactopyranose units. Two primary types are identified: carrageenans, which feature a 4-linked α-galactose with a dextro-rotatory (D-) configuration; and agarans, with a levorotatory (L-) 4-linked α-galactose component [75,76,77]. Carrageenan has shown antiviral properties, because of its property of disrupting the interaction between the virus and host cell receptors and preventing viral entry. Iota-carrageenan, a sulfated polysaccharide from seaweed, at low concentrations (4 μg/mL) reduced cell death caused by the SARS-CoV-2 virus, while high concentrations of κ and λ-carrageenans (400 μg/mL) offered only partial suppression, indicating that carrageenan/iota-carrageenan may be more effectively used during the initial stages of infection [78]. An iota-carrageenan nasal spray effectively treated common cold symptoms linked to human coronaviruses, reducing symptom recurrence and improving viral clearance compared to placebo treatments [78]. A nasal spray combining xylometazoline hydrochloride and carrageenan has alleviated nasal congestion and protected the respiratory mucosa from viruses [79]. Lozenges containing iota-carrageenan have inactivated viral glycoproteins in the mouth, blocking viral effects. Iota-carrageenan also neutralized a SARS-CoV-2 spike pseudotyped lentivirus, suggesting its potential efficacy in COVID-19 prevention [80]. Marine-derived saponins from sea cucumbers [81] can boost immunization [82] and could be incorporated into vaccine formulations as adjuvants. Other adjuvants could come from marine lipids found in microalgae that can form stable emulsions that increase the bioavailability of antigens and facilitate their uptake by immune cells [56,59]. Molecules of marine origin could also serve as therapeutics or therapeutic support. For example, infection with several viruses including SARS-CoV-2 infection triggers the release of reactive oxygen species (ROS), increasing susceptibility to further infection. Therefore, marine algae-derived compounds with strong antioxidant properties like fucoxanthin and fucosterol (Figure 1) could counter oxidative damage, reduce overall oxidative stress, and protect immune cells and host tissues during active infection.

More specifically, fucoxanthin from *Sargassum siliquastrum* reduces DNA damage and enhances antioxidant enzyme levels, while fucosterol boosts cellular antioxidant enzymes and protects human hepatic cells from oxidative stress [83,84]. Table 1 summarizes published information on marine-derived adjuvants and antioxidants and presents future directions, emphasizing the potential of these compounds in antiviral treatments and the need for ongoing research to better understand their mechanisms and full therapeutic potential.

Other marine natural products [85] from sea urchins, sea cucumbers, sponges, soft corals, and microalgae exhibited various bioactive properties, including antioxidant, anti-inflammatory, anti-cancer, and immune enhancement properties. Moreover, marine natural products have yielded antimicrobials against coronavirus (SARS-CoV-2 and its variants), tuberculosis, *H. pylori*, and HIV, making them promising resources for managing several pathologies [86]. The anti-inflammatory mechanisms of marine natural products in SARS-CoV-2 infection are particularly notable, as they offer therapeutic benefits with fewer cardiovascular side effects compared to other chemical agents used in COVID-19 treatment. With their ability to target multiple pathways involved in immune regulation and inflammation inhibition, these products hold significant potential for further clinical applications, making the sustainable development of marine ecosystems critical for future biomedical advances [86]. Dendritic cells (DCs) bridge innate and adaptive immunity by presenting foreign antigens to T-cells and secreting cytokines that activate and coordinate the adaptive response and lead to long-lasting responses [87]. Activating DCs is considered a crucial strategy to improve vaccination efficacy. In this context, a novel marine-derived immunomodulatory sulfolipid, β-SQDG18, appeared to be capable of priming human DCs independent of TLR2/TLR4 and to trigger an effective immune response in vivo [88]. β-SQDG18 promotes DC maturation and increases expression of MHC II molecules and the co-stimulatory CD83 and CD86 proteins, along with pro-inflammatory cytokines such as IL-12 and INF-γ, which are necessary for antigen presentation to CD4+ T-cells. Mice vaccinated with ovalbumin combined with β-SQDG18 (1:500) generated anti-ovalbumin Ig titers comparable to conventional adjuvants. In a melanoma model, vaccination of C57BL/6 mice with β-SQDG18-adjuvanted hgp10 peptide induced a protective response, slowing tumor growth and extending survival [88].

### 3.2. Bioactive Compounds from Marine Sources: Antiviral Potential and Immunomodulatory Effects

**Figure 2:**
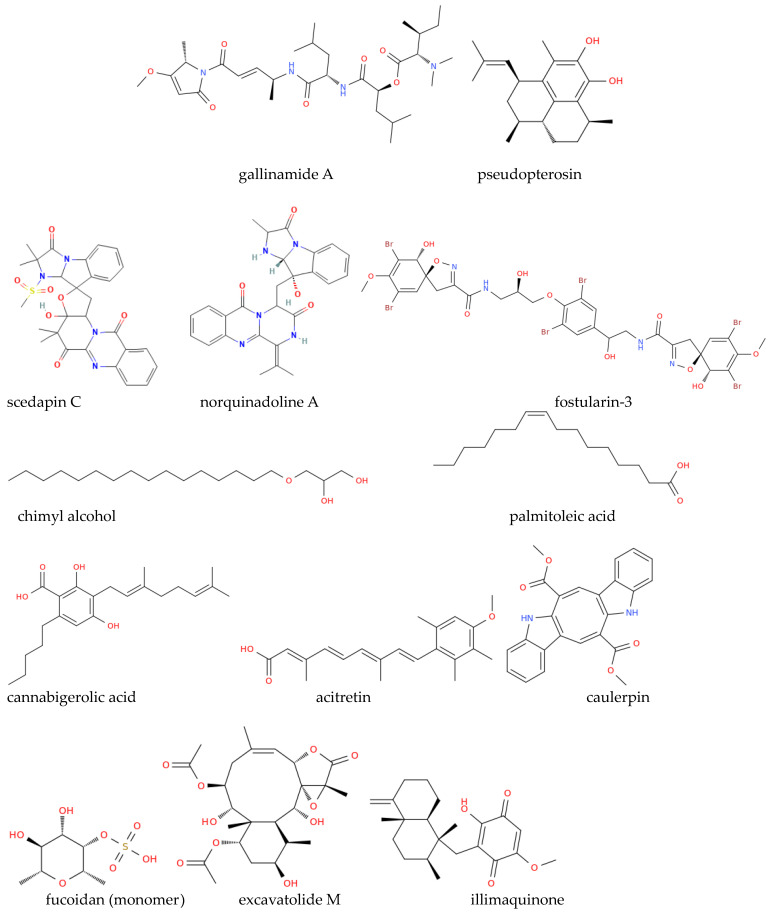

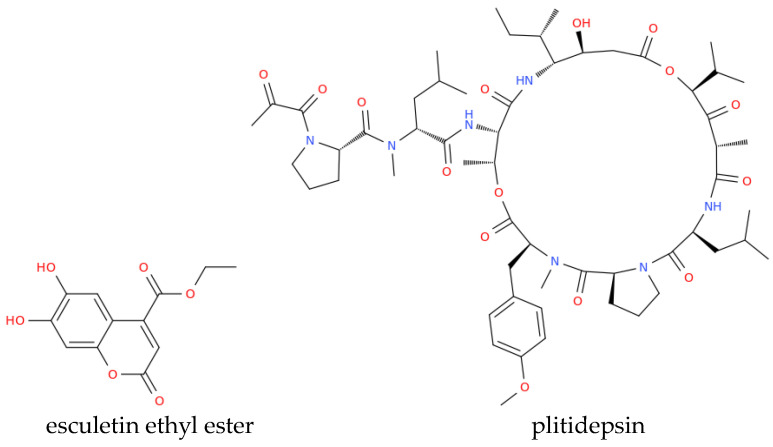
Structure representation of some of the marine compounds investigated as anti-COVID drugs discussed by Asif et al. [89].

Marine-derived bioactive molecules, such as griffithsin, and plitidepsin, a cyclic depsipeptide isolated from *Aplidium albicans* (Figure 2), are currently undergoing clinical trials to assess antiviral efficacy [89]. Several marine phytochemicals can bind with key SARS-CoV-2 drug targets and possess anti-inflammatory and immunomodulatory effects, potentially mitigating COVID-19 complications. The structures of the anti-COVID compounds mentioned in this section are shown in Figure 2 to illustrate the diversity of marine-derived molecules. These structures encompass different chemical classes of substances, demonstrating the extensive arsenal available from marine sources to combat coronaviruses, particularly SARS-CoV-2. This diversity should serve to inspire new semi-synthetic and clinical studies aimed at optimizing existing natural structures or their combined use in therapy to achieve stronger activities against variants of SARS-CoV-2 or new coronaviruses that may emerge in the future. Molecular docking [90] and MD simulation studies by Quimque et al. evaluated the binding affinity of marine alkaloids scedapin C and norquinadoline A (Figure 2) against SARS-CoV-2 targets [89]. Both compounds demonstrated a high binding affinity for PLpro, a crucial enzyme in SARS-CoV-2 replication and immune response suppression, with the same study suggesting that scedapin C and norquinadoline A may inhibit PLpro, potentially blocking viral replication and activating immune responses. The efficacy of the marine alkaloid fostularin-3, chimyl alcohol, palmitoleic acid, cannabigerolic acid, and acitretin against SARS-CoV-2 was also investigated. Molecular docking and MD simulations revealed that fostularin-3 forms hydrogen bonds and hydrophobic interactions with residues in the Mpro enzyme, a key target for anti-SARS-CoV-2 drugs [89]. Caulerpin, an alkaloid from various marine algae, showed strong binding to the SARS-CoV-2 main protease (3CLpro) and favorable pharmacokinetic properties, while molecular docking indicated its potential to halt the virus’s life cycle and its anti-inflammatory effects by downregulating pro-inflammatory cytokines involved in COVID-19’s hyperinflammatory phase. Furthermore, C-phycocyanin, a pigment from the blue-green algae *Arthrospira platensis* [91], has shown potential to inhibit SARS-CoV-2 non-structural proteins (nsp-8, nsp-7, and nsp-12), with docking studies suggesting its ability to block nsp-12, a key protein in viral replication. Moreover, nutrient-rich spirulina has been suggested to enhance immune function and reduce inflammation, which could be beneficial for COVID-19 management [89]. Marine polyphenols, including quercetin from brown algae (genus *Sargassum*), exhibit antiviral properties. Consistent with this observation, different studies indicate that quercetin-based treatments may alleviate respiratory symptoms and inflammation associated with COVID-19 in vivo [89]. However, quercetin is classified as a PAINS (pan-assay interference compound), which refers to molecules that non-specifically interact with multiple biological targets and can lead to misleading results in drug screening assays [92,93]. Due to its promiscuous binding behavior, quercetin’s antiviral effects may not always stem from specific interactions with viral proteins, and thus, further studies are needed to confirm its therapeutic potential while accounting for these non-specific effects [94].

**Figure 3:**
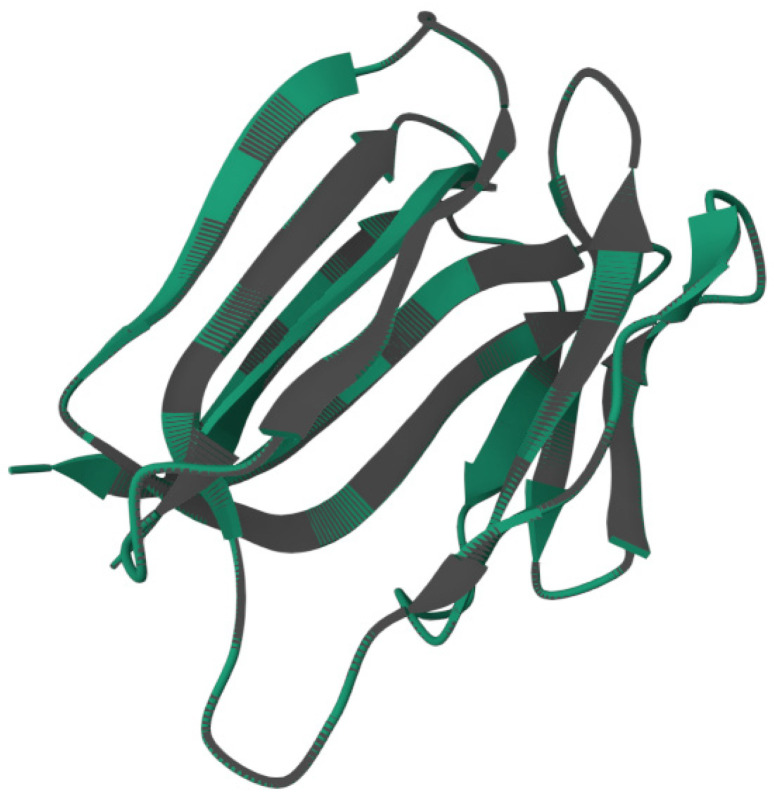
Three-dimensional view of the protein griffithsin from *Griffithsia* sp. The picture is freely available at the link https://www.rcsb.org/3d-view/3LL2/1 (accessed on 9 September 2024).

Studies by Song et al. [95] assessed the anti-SARS-CoV-2 activities of the above-mentioned marine sulfated polysaccharides, including sea cucumber sulfated polysaccharide (SCSP), fucoidan from brown algae, iota-carrageenan from red algae, and chondroitin sulfate C from sharks. SCSP demonstrated the strongest inhibitory activity, potentially blocking viral entry into host cells, whereas fucoidan has shown promise in reducing inflammation and enhancing vaccine responses, warranting further research [95]. Molecular docking studies evaluated briarane-type diterpene excavatolide M from gorgonian (*Briareum excavatum*) for its ability to bind SARS-CoV-2 TMPRSS2 and the potential of illimaquinone, a marine sponge metabolite, against SARS-CoV-2 target proteins, including papain-like protease, compared to standard antiviral drugs [89]. An in silico study identified esculetin ethyl ester from marine sponge *Axinella* cf. *corrugata* as a compound with a strong binding affinity to SARS-CoV-2 protease N3, while seaweed lectins, including griffithsin (Figure 3) from *Griffithsia* sp., inhibited various enveloped viruses in silico [89]. Griffithsin has shown the ability to block SARS-CoV spike glycoprotein and prevent viral entry into host cells, which recalled clinical trials aimed at investigating its potential against HIV and SARS-CoV-2.

Overall, we can conclude that marine-derived bioactive molecules have promising potential applications for combating COVID-19. Some, like spirulina (*Arthrospira platensis*) are already in use [96], while others, such as griffithsin and plitidepsin (Figure 2), have entered clinical evaluation. Ongoing studies are crucial to further explore their therapeutic potential and determine their efficacy against viruses from the beta coronavirus family [89].

Table 2 summarizes marine-derived compounds identified through molecular docking studies and other approaches [89] for their specific interactions with SARS-CoV-2 proteins. Scedapin C, norquinadoline A, and fostularin-3 show potential in inhibiting key viral enzymes such as PLpro and Mpro, which are crucial for viral replication. Additionally, *caulerpin* and *C-phycocyanin* exhibit anti-inflammatory properties alongside their ability to block viral replication. Others, such as marine-derived polysaccharides like fucoidan and iota-carrageenan, target viral entry, while *griffithsin* shows promise in blocking the spike glycoprotein. These compounds offer potential as therapeutic agents against COVID-19 by targeting specific proteins involved in the virus’s life cycle.

Beyond predictive in silico studies, preclinical trials have also highlighted marine compounds’ therapeutic and prophylactic potential [107]. Various concentrations of fucoidan, specifically RPI-27 and RPI-28, extracted from *Saccharina japonica* have demonstrated antiviral activity against SARS-CoV-2 in Vero cells. RPI-27 significantly inhibited infection (EC_50_ = 0.08 μM), showing greater efficacy compared to RPI-28 (EC_50_ = 1.2 μM). Carrageenans, may prevent SARS-CoV-2 entry through the nasal cavity by interacting with the virus’s positively charged membrane. Additionally, plitidepsin showed 90% inhibitory activity at 0.88 nM against SARS-CoV-2, far exceeding the efficacy of the antiviral remdesivir [107]. Another marine compound, gallinamide A, from the cyanobacteria *Schizothrix*, inhibited cathepsin L and significantly reduced viral load. Furthermore, the above-mentioned lectin griffithsin has shown high specificity for viral glycoproteins, effectively inhibits early-stage viral infection in a dose-dependent manner, and completely protected SARS-CoV-2-infected rats treated with it (100% survival). The already discussed esculetin ethyl ester has also been shown to inhibit the SARS-CoV 3CLpro/Mpro enzyme, further highlighting the antiviral potential of marine-derived compounds [107]. Remarkably, marine natural products [108,109,110,111,112] are particularly advantageous over synthetic chemicals [113] in treating COVID-19 due to fewer adverse cardiovascular effects. An example is pseudopterosin from the Caribbean sea whip, which has been used to prevent skin irritation [114]. Marine organisms have endured for millions of years, developing metabolites that enable them to survive harsh conditions. Some of these metabolites, found in microalgae, exhibit antiviral properties and help combat various diseases, with microalgae being abundant in amino acids, saccharides, vitamins, minerals, and metabolites that support immune health. *Cyanophyta* algae, particularly spirulina, demonstrate potent antiviral activity. Clinical trials with spirulina involving 30 patients showed promising results in reducing viral infections [115,116]. Marine microalgal polysaccharides like naviculan from *Navicula directa* (*Bacillariophyta*) and polysaccharides A1 and A2 from dinoflagellate *Margalefidinium polykrikoides* (formerly *Cochlodinium polykrikoides*) have shown antiviral efficacy against HIV-1 and influenza type A virus [117]. Nutrient-rich microalgae such as *Chlorella* (*Chlorophyta*) have boosted immune responses and are used in cancer treatments. In particular, hydrophilic extracts from *Chlorella* have shown potential for lowering blood sugar, reducing hyperlipidemia, and improving immunity, while the green microalga *Haematococcus lacustris* (formerly *Haematococcus pluvialis*) is rich in astaxanthin [118], which enhances IgA, IgG, and IgM immunoglobulin production by activating T-helper cells, improves immune response via NK cells, and reduces stress-related inflammation [119]. *Navicula directa* has shown antiviral activity against herpes simplex virus 1 (HSV-1) and herpes simplex virus 2 (HSV-2). Additionally, *A. platensis* has exhibited antiviral effects against mumps, influenza, HIV, polio, and measles viruses, while *Nostoc flagelliforme* (*Cyanobacteria*) has been effective against influenza A and herpes viruses [117].

### 3.3. Other Marine-Derived Antimicrobials

**Figure 4:**
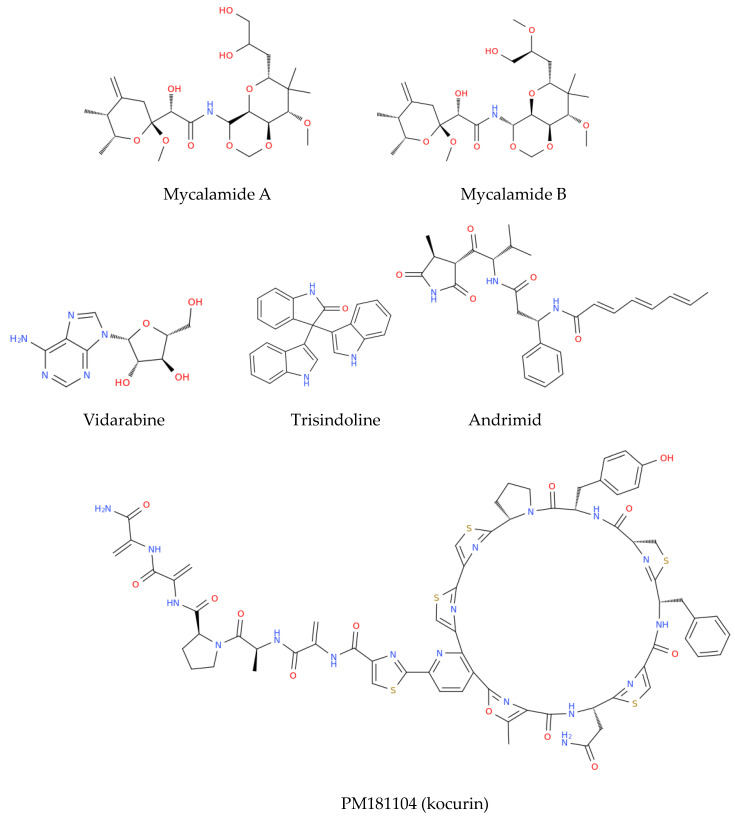

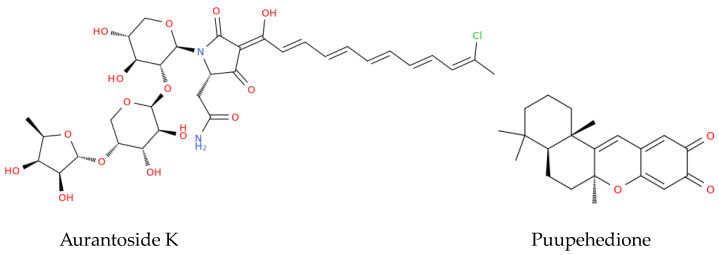
Structure representation of the marine-derived antimicrobials discussed in Section 3.3.

With the term “antimicrobials”, we refer to a broad category of substances that kill or inhibit the growth of microorganisms. This includes antibiotics, antifungals, antivirals, and antiparasitics. Several noteworthy antimicrobials have been identified from marine sources, particularly sponges and their associated bacteria (Figure 4, Table 3).

Mycalamide A and Mycalamide B, antiviral compounds derived from a New Zealand sponge (*Mycale* sp.), were first reported for their antiviral activity in the late 1980s. These compounds also exhibit antitumor properties and inhibit protein synthesis, showcasing activity against murine coronavirus A59, HSV, and polio viruses. Mycalamide A has additionally demonstrated the ability to inhibit influenza virus replication [120]. Vidarabine, a purine nucleoside analogue isolated from the sponge *Cryptotethya crypta*, gained prominence as one of the first successful antiviral agents licensed in 1977. Despite being largely replaced by acyclovir due to its toxicity and poor solubility, vidarabine remains significant for its effectiveness against herpes simplex virus, cytomegalovirus, and varicella zoster virus (VZV) [121]. It is particularly valuable for treating acyclovir-resistant strains of HSV and VZV and is still used in ophthalmic procedures in the European Union [122]. Trisindoline, identified in extracts of *Callyspongia siphonella*, is responsible for significant antibacterial and cytotoxic activities. Compounds such as 5-bromotrisindoline and 6-bromotrisindoline were isolated through bioactivity-guided fractionation, exhibiting effective antibacterial properties against *S. aureus* and *Bacillus subtilis* [123]. Andrimid, a peptide antibiotic originating from *Hyatella* sp., displays a broad spectrum of antibacterial activity against both Gram-positive and Gram-negative bacteria, including methicillin-resistant *S. aureus* (MRSA) and bacterial pathogens including *Salmonella enteritidis*, *Vibrio harveyi*, and *Yersinia ruckeri* [124]. This compound was isolated from a bacterial strain associated with the sponge and has also been sourced from *Pseudomonas fluorescens*. The widespread occurrence of andrimid among delta-Proteobacteria suggests a role for horizontal gene transfer in its dissemination [125]. PM181104, a thiazolyl cyclic peptide antibiotic derived from the marine sponge *Spirastrella inconstans* var. *digitata* exhibits potent antibacterial activity against MRSA and other bacterial pathogens. Characterized by various analytical methods, PM181104 effectively inhibits bacterial protein synthesis and has demonstrated minimal inhibitory concentrations (MIC) against both resistant and sensitive strains of *S. aureus* and *Enterococcus* sp. Notably, it has shown non-toxicity to mammalian cell lines, positioning it as a promising therapeutic agent. In in vitro testing against a variety of organisms, PM181104 revealed strong inhibitory action on *S. aureus* with an MIC range of 0.008 to 2.048 µg/mL, and its efficacy was comparable to that of standard antibiotics in various in vivo models of infection [126]. Aurantoside K, sourced from *Melophlus* sp., has shown inhibitory activity against various pathogenic fungi, including wild-type and amphotericin B-resistant *C. albicans*. Its antifungal efficacy extends to *Cryptococcus neoformans*, demonstrating potential against a range of fungal pathogens [127].

Marine-derived antimicrobials hold significant promise for enhancing vaccine development and efficacy. Notably, compounds such as Puupehedione, isolated from the Verongid sponge, demonstrate diverse beneficial properties, including antimicrobial, and immunomodulatory activities [120,128] that enhance the immune response, potentially improving vaccine effectiveness. Furthermore, specific marine compounds could be incorporated into formulations to target pathogens directly or strengthen immune recognition.

## 4. Marine-Derived Delivery Systems for Vaccines

**Scheme 2:**
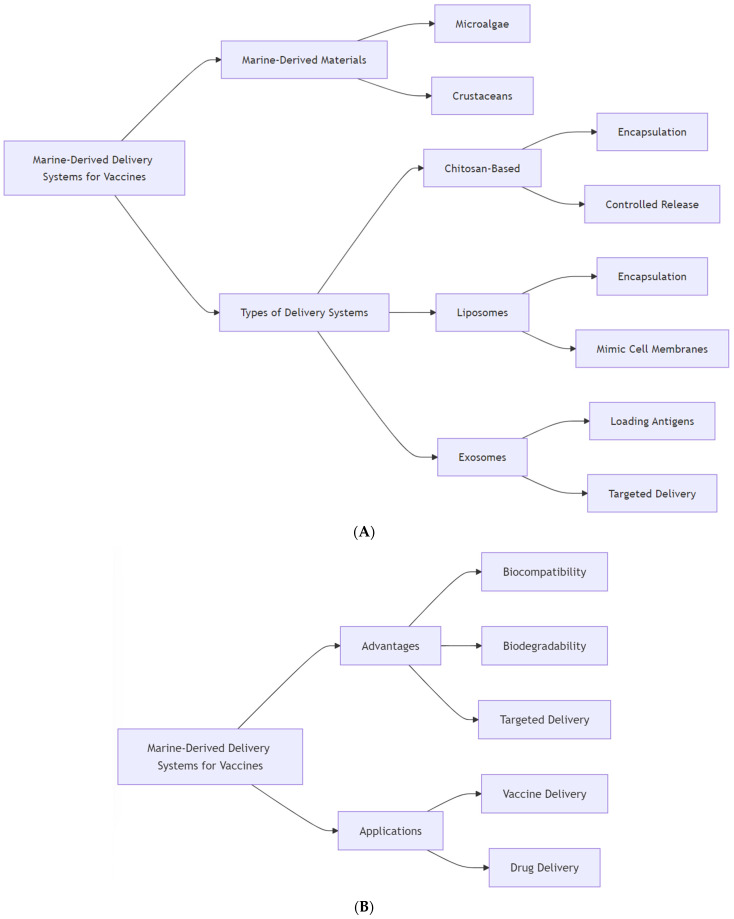
Flowcharts of marine-derived delivery systems for vaccines illustrate the key components and advantages of marine-derived materials used in vaccine delivery systems. (**A**): Highlights the types of marine-derived materials, including microalgae and crustaceans, and outlines the various delivery systems such as chitosan-based systems, liposomes, and exosomes. It details their specific applications, including encapsulation and controlled release mechanisms. (**B**): Focuses on the advantages of marine-derived delivery systems, emphasizing biocompatibility, biodegradability, and targeted delivery capabilities. Additionally, it outlines the applications of these systems in both vaccine and drug delivery.

Marine biodiversity [129,130,131] promises innovative vaccine delivery systems (Scheme 2) with potential for high biocompatibility, biodegradability, and ability to be engineered for targeted delivery, making them ideal candidates for modern vaccine development. One prominent example is chitosan [132], a biopolymer derived from the exoskeletons of crustaceans, which has gained attention for its ability to form nanoparticles and encapsulate antigens. Chitosan-based delivery systems can protect the vaccine’s active ingredients, enhance mucosal immunity, and provide controlled release, thereby improving the vaccine’s effectiveness. Additionally, liposomes and other lipid-based carriers, sourced from marine organisms, have been utilized to encapsulate and deliver vaccine antigens with high efficiency. These carriers can be engineered to mimic natural cell membranes, facilitating the fusion with target cells and enhancing antigen presentation. Moreover, marine-derived exosomes [133], extracellular vesicles naturally released by cells [134], have shown promise as novel delivery vehicles. These vesicles can be loaded with vaccine antigens and possess intrinsic properties that aid in targeted delivery to immune cells, enhancing the immune response.

As previously mentioned, chitosan, a biopolymer sourced from different marine organisms [135,136] has gained significant interest in the realm of drug delivery systems. It has been explored for its potential in various applications, including the development of chitosan composites and nanoparticles for a range of drug delivery methods [137]. Other marine polysaccharides, such as fucoidan, have also been used in drug and vaccines delivery systems. In particular, fucoidan-based nanoparticles have potential as vaccine delivery systems. In this context, fucoidan-N-(2-hydroxy-3-trimethylammonium) propylchitosan (FUC-HTCC) nanoparticles were evaluated as adjuvants for the anthrax vaccine (AVA) [138,139]. These nanoparticles, which combine the marine polysaccharide fucoidan with a modified form of chitosan, were shown to be effective in enhancing vaccine efficacy. When used in conjunction with the anthrax vaccine, the FUC-HTCC nanoparticles significantly increased the titer of protective IgGs, indicating a stronger humoral immune response. Moreover, their uptake by dendritic cells, which are crucial for initiating immune responses, was notably efficient. The enhanced immune response led to superior protection against anthrax toxin in mouse models, demonstrating their potential for vaccine delivery and as adjuvants [138]. Nano-based drug delivery systems, showcasing the potential of chitosan, along with other marine-derived materials, have been applied as nanocarriers for anticancer drug delivery systems, offering improved efficiency and targeting [140]. Studies have shown that chitosan nanoparticles have advanced parenteral drug delivery, oral drug administration, non-viral gene delivery, and vaccine delivery [141,142], particularly for mucosal vaccines [132]. The adjuvant potential of chitosan is largely due to its mucoadhesive nature, which allows it to interact with negatively charged mucosal surfaces. This feature enables non-invasive, needle-free vaccine administration, promoting both systemic and mucosal immune responses. The chitosan free amino groups contribute to controlled antigen release, transfection, enhancement of permeation, and inhibition of efflux pumps, which are critical for effective vaccine delivery. Chitosan’s ability to enhance transmucosal absorption is especially advantageous for vaccines, as it facilitates antigen uptake across mucosal surfaces, thereby improving immune responses [132]. When used as an adjuvant in mucosal vaccines, chitosan has been shown to induce robust antibody and T-cell responses [143,144]. To optimize its solubility and broaden its medical applications, chitosan can be modified through acylation, alkylation, and sulfation that improve its effectiveness as a vaccine adjuvant, particularly when formulated into micro- or nanoparticles. Chitosan microspheres are especially effective, as they provide controlled release of antigens, outperforming commonly used materials like polylactic acid (PLA) and poly (lactic-co-glycolic) acid (PLGA) in this role [132]. This controlled release helps eliminate the need for repeated vaccine doses, a significant advantage over conventional vaccine formulations. Overall, chitosan’s unique properties make it a highly effective material for enhancing vaccine delivery and immune responses.

**Figure 5:**
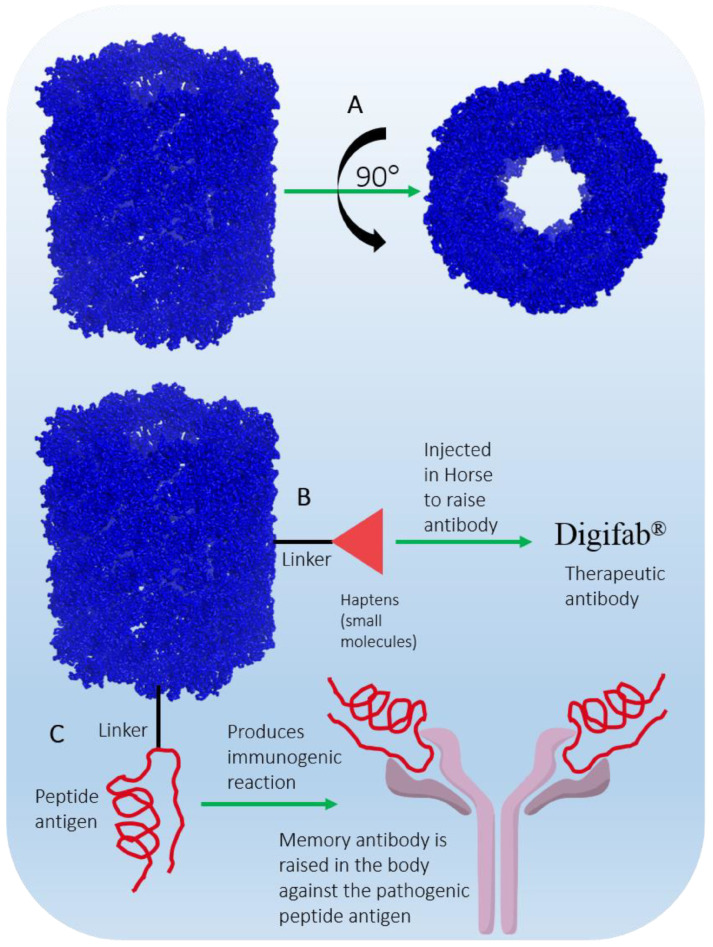
(**A**) KLH is depicted in a barrel-shaped structure, visualized using cryo-electron microscopy with 3D coordinates (PDB ID 4BED). (**B**) KLH functions as a hapten conjugate, allowing it to bind small molecules. (**C**) KLH is utilized as a carrier molecule in vaccines, aiding in the delivery and presentation of antigens for an enhanced immune response. This picture is freely available at the link https://www.mdpi.com/1660-3397/20/8/528 (accessed on 9 September 2024) [145].

Multimeric forms of keyhole limpet hemocyanin (KLH) represent another example of marine-derived systems for vaccine delivery (Figure 5). KLH, a large barrel-shaped metalloprotein found in the hemolymph of the marine mollusk *Megathura crenulata*, plays a crucial role in the development of therapeutic agents targeting cancer and other immune-related diseases. While KLH itself is not a drug, its ability to stimulate a targeted immune response has made it invaluable in immunological research and the immunopharmaceutical industry for over 50 years. KLH exists in two isoforms, KLH1 and KLH2, with monomeric molecular weights of 390 kDa and 360 kDa, respectively. Its structure consists of 20 monomers, each containing 7–8 functional domains that bind two copper ions (Cu^2+^) and one oxygen molecule. The didecameric (20-unit) assembly of KLH is highly glycosylated, making it ideal for hapten conjugation and as a carrier molecule in vaccines. Unlike synthetic alternatives, KLH cannot be easily produced in a laboratory due to its intricate structure and size, so it is commercially extracted from the mollusk’s hemolymph. Despite its potent immunogenicity, KLH does not trigger harmful immune reactions in humans, making it a safe and effective immunomodulatory agent. It has been used extensively in both experimental and clinical settings, underscoring its value in advancing therapeutic approaches and vaccine delivery systems [145]. Overall, marine ecosystems, with their abundant biodiversity, provide a wealth of materials for innovative vaccine delivery systems. From chitosan’s mucoadhesive properties to fucoidan-based nanoparticles and KLH, marine-derived substances enhance immune responses and improve antigen delivery. These natural materials offer biocompatibility, biodegradability, and targeted delivery capabilities, making them invaluable in the development of more effective and accessible vaccines. Their unique properties promise to revolutionize vaccine technology by improving stability, immune activation, and precision in targeting specific immune pathways.

## 5. Marine-Derived Antineoplastics

As mentioned previously, several marine-derived compounds including polysaccharides, peptides, proteins, phenolic compounds, and alkaloids are currently being studied for their use as possible cancer treatments. Their origins are from several taxonomic groups, such as brown algae, marine fungi, polychaete worms, sponges, and tunicates [126], highlighting the need to preserve the marine biodiversity. One such compound, trabectedin, paved the way for marine antineoplastics as the first derived from a marine animal, the Caribbean sea squirt *Ecteinascidia turbinata*, a tunicate. Trabectedin is now used to treat soft tissue sarcoma [127]. In 2022, a phase 3 clinical trial showed that a combination of trabectedin and doxorubicin was more effective in treating metastatic leiomyosarcoma than the first-line treatment of doxorubicin alone [128]. Additionally, trabectedin and its structural derivative lurbinectedin are under examination for treatment of soft tissue sarcoma and relapsed ovarian cancer in combination with doxorubicin. Lurbinectedin was approved after a phase 2/3 trial in 2020 for the treatment of small cell lung cancer that is resistant to platinum-based chemotherapy [129,130]. Several derivatives from various marine invertebrates exhibit anticancer, but also anti-parasitic, antihypertensive, antifungal, antiviral, antibacterial, and immune-modulatory activities [146,147]. Extracts from mollusks also show antioxidant, anticancer, anti-infective, and cardiovascular protective effects. Sea cucumber protein hydrolysates are particularly noted for their ability to reduce reactive oxygen species accumulation and scavenge free radicals [148]. Other bioactive substances being evaluated as potential cancer inhibitors include the cryotin enzyme from shrimp and protamex from snow crabs, as well as antioxidant neutrase from sea urchins [114].

## 6. Conclusions

The exploration of marine-derived substances represents a frontier in medical science that holds immense promise for human health. Marine natural products significantly contribute to vaccine development. The unique ability of microalgae to produce complex antigens and bioactive compounds under controlled conditions underscores their critical role in creating sustainable and effective vaccines suitable to modern technologies. Beyond vaccines and biotechnology to express complex antigens in microalgae, the ocean’s bounty includes powerful adjuvants, advanced delivery systems, and immunomodulatory agents that can significantly enhance immunological responses and vaccine effectiveness. Novel antimicrobial compounds may enhance vaccine performance. Also, marine organisms have provided numerous antiviral compounds. Marine bivalves, for instance, are rich in alkaloids, terpenoids, steroids, polysaccharides, and peptides, all of which have potential antiviral properties [146,147]. Remarkably, marine compounds feature excellent unique biocompatibility, biodegradability, and targeted delivery capabilities that promise to revolutionize vaccine technology and contribute to broader human health. They improve stability, enhance immune activation and the targeting of specific immune pathways, making them invaluable in the quest for better immunization strategies. Many of these bioactives exhibit additional desirable properties, including antioxidant and cardiovascular normalization, that improve their tolerability and desirability as drug. Marine invertebrates like mollusks, sponges, crustaceans, and echinoderms are excellent sources of bioactive compounds, including peptides, phenols, steroids, alkaloids, terpenoids, and strigolactones [12]. Bioactives from the *Mytilidae* family of mussels include proteins, lipids, carbohydrates, polyunsaturated fatty acids, and iodine, all known for their antimicrobial and anti-inflammatory properties. Remarkably, marine-derived compounds are emerging as particularly promising in oncology [149,150]. While promising for human health applications, marine-derived products also have certain disadvantages. A primary concern is their potential toxicity, as seen with vidarabine, which limits their safe therapeutic use due to harmful side effects. Additionally, sustainability may become an issue when the harvesting of marine organisms can threaten marine ecosystems and biodiversity, especially for slow-growing species. The extraction and isolation of these compounds are often complex and expensive due to their low natural concentrations, making large-scale production difficult. Furthermore, regulatory challenges arise because these novel compounds often lack comprehensive safety data, leading to prolonged approval processes. Finally, many of these substances have mechanisms of action that are not yet fully understood, complicating their development and integration into existing therapeutic frameworks while posing risks of unforeseen side effects. These marine innovations are not only paving the way for more effective vaccines but also contributing to a more comprehensive approach to disease prevention and human health. In the quest to rapidly broaden the library of safe bioactives to support human health, new applications of model organism research may accelerate progress toward the characterization of their biological properties, mechanism of action, and toxicological properties. The fruit fly *Drosophila melanogaster*, for example, is an excellent whole-animal model suited to perform rapid miniaturized pharmacological testing. Despite substantial morphological differences, flies and humans share about 75% of disease-related genes and, crucially, also share their higher order connection in pathways [151,152]. Conservation extends to key toxicological pathways, which makes it possible to leverage the month-long lifespan of the fly to detect even trace level activation during chronic exposure to predict possible health consequences in a fraction of the time needed for equivalent studies in vertebrates [153]. Similarly, miniature drug assays using analytical amounts of testing compounds and wild-type or ad hoc mutant fly lines, or “humanized” flies expressing specific human proteins, can yield information on biological activity, dose-response effects, and, when combined with extensive *Drosophila* genetic resources and “omics” approaches, also mechanism of action [152,154,155,156]. *Drosophila* does not reproduce all the complexities of human physiology, e.g., it only has innate immunity. However, *Drosophila* can be used effectively to probe the effects of both single compounds and mixtures on development and on the fundamental pathways conserved throughout evolution, economizing time and costs. Such studies could enable rapid prioritization of promising bioactives [157] for escalation to longer and costlier vertebrate and clinical research studies, and they could also provide unique insights into molecular mechanisms and toxicity. Collaborative efforts among marine biologists, pharmacologists, and immunologists are vital to unraveling the mechanisms by which marine compounds modulate immune response and developing innovative strategies to bolster vaccine performance. Integrating marine-derived antimicrobials into vaccine formulations may improve stability and effectiveness, which is particularly important in the face of emerging infectious diseases. As the exploration of oceanic resources continues, it is crucial to protect marine ecosystems to sustain their biodiversity and health and ensure the continued availability of these valuable compounds. Marine biotechnology holds immense promise for advancing medical science, and ongoing research and technological advancements are vital to unlock the full therapeutic potential of marine-derived compounds. By investing in marine biotechnology, we can enhance our ability to develop effective vaccines and treatments, contribute to a more comprehensive approach to disease prevention and immune health, and emphasize the importance of sustainable practices to safeguard our oceanic resources.

## Figures and Tables

**Table 1:** Overview of marine-derived antioxidants and adjuvants for vaccine development and their potential applications.

Marine-Derived Compound (Type)	Application/Effect
**Fucoidan** (adjuvant from brown seaweed)	Stimulates both humoral and cellular immune responses, enhancing vaccine efficacy.
**Saponins** (adjuvant from sea cucumbers)	Enhances antigen presentation, promoting a robust immune response in vaccines.
**Microalgae-derived lipids** (adjuvant)	Creates stable emulsions, improving bioavailability and offering immunomodulatory properties.
**Carrageenan** (antiviral polysaccharide)	Exhibits antiviral activity, especially against SARS-CoV-2, used in nasal sprays and hygiene products.
**Iota-carrageenan** (antiviral polysaccharide)	Reduces symptoms of common cold and inactivates viral glycoproteins.
**Fucoxanthin** (antioxidant from Sargassum siliquastrum)	Mitigates DNA damage, enhances antioxidant enzyme levels, and protects cells from oxidative stress.
**Fucosterol** (antioxidant from marine sources)	Boosts cellular antioxidant defenses and protects human hepatic cells from oxidative damage.

**Table 2:** Marine-derived compounds targeting SARS-CoV-2 proteins and their potential activities [89].

Compound	Marine Source	SARS-CoV-2 Protein Target	Potential Activity	Ref.
Scedapin C	*Scedosporium apiospermum*	PLpro	Inhibits viral replication and activates immune responses by blocking PLpro activity	[97]
Norquinadoline A	*Scedosporium apiospermum*	PLpro	Inhibits PLpro, potentially blocking viral replication and boosting immune response	[97]
Fostularin-3	*Alpysinidae*	Mpro	Forms hydrogen bonds and hydrophobic interactions with Mpro, potentially inhibiting virus	[98]
Caulerpin	*Caulerpa racemosa*	Mpro	Inhibits virus life cycle, anti-inflammatory properties by down-regulating cytokines	[99,100]
Quercetin	Brown algae (*Sargassum genus*)	ACE2 receptor	Disrupts ACE2 receptor interactions, reduces respiratory symptoms and inflammation	[101]
Fucoidan	Brown algae	Viral entry proteins	Inhibits the S-glycoprotein of SARS-CoV-2 and disrupts the ERK signaling pathway. Anti-inflammatory and enhances vaccine response	[95]
Iota-carrageenan	Red algae	Viral entry proteins	Potential to block viral entry	[102]
Chondroitin sulfate C	Sharks	Viral entry proteins	Potential to block viral entry	[95]
Excavatolide M	Gorgonian (*Briareum excavatum*)	TMPRSS2	Shows potential to inhibit TMPRSS2	[103]
Illimaquinone	Marine sponge	PLpro	Inhibits papain-like protease, antiviral potential	[104]
Esculetin ethyl ester	Marine sponge (*Axinella* cf. *corrugata*)	N3 protease	Strong binding affinity to N3 protease	[105]
Griffithsin	*Griffithsia* sp. (seaweed)	Spike glycoprotein	Blocks spike glycoprotein, preventing viral entry into host cells	[105,106]

**Table 3:** Marine-derived antimicrobials discussed in the Section 3.3.

Marine-Derived Antimicrobial	Marine Source	Activity	Organism(s) Active Against	References
Mycalamide A & B	New Zealand sponge (*Mycale* sp.)	Antiviral, antitumor, protein synthesis inhibition	Murine coronavirus A59, HSV, Polio, Influenza virus	[120]
Vidarabine	Sponge (*Cryptotethya crypta*)	Antiviral	Herpes simplex virus (HSV), Cytomegalovirus, Varicella zoster virus (VZV)	[121,122]
Trisindoline	Sponge (*Callyspongia siphonella*)	Antibacterial, cytotoxic	*S. aureus*, *Bacillus subtilis*	[123]
Andrimid	Sponge (*Hyatella* sp.) and bacteria (*Pseudomonas fluorescens*)	Broad-spectrum antibacterial	Methicillin-resistant *Staphylococcus aureus* (MRSA), *Salmonella enteritidis*, *Vibrio harveyi*, *Yersinia ruckeri*	[124,125]
PM181104	Marine sponge (*Spirastrella inconstans* var. *digitata*)	Antibacterial, protein synthesis inhibition	MRSA, *Enterococci*, *S. aureus* (resistant and sensitive strains)	[126]
Aurantoside K	Sponge (*Melophlus* sp.)	Antifungal	Amphotericin B-resistant *C. albicans*, *Cryptococcus neoformans*, *A. niger*, *Penicillium* sp., others	[127]
Puupehedione	Verongid sponge	Antitumor, anti-angiogenic, antimicrobial, immunomodulatory	Various microbial pathogens	[120,128]
